# Clear cell sarcoma of the kidney distinguished from synovial sarcoma using genetic analysis: a case report

**DOI:** 10.1186/s13104-015-1100-5

**Published:** 2015-04-08

**Authors:** Masahito Hirose, Kentaro Mizuno, Hideyuki Kamisawa, Hidenori Nishio, Yoshinobu Moritoki, Kenjiro Kohri, Yutaro Hayashi

**Affiliations:** Department of Nephro-urology, Nagoya City University Graduate School of Medical Sciences, 1 Kawasumi, Mizuho-cho, Mizuho-ku, Nagoya, 467-8601 Aichi Japan

**Keywords:** Gene analysis, Sarcoma, Pediatric renal tumor

## Abstract

**Background:**

The most common pediatric renal neoplasm is Wilms tumor, but clear cell sarcoma of the kidney or synovial sarcoma of the kidney are also sometimes encountered. Accurate pathological diagnosis is important, because adjuvant therapies including chemotherapy and radiotherapy differ according to the pathological type.

**Case presentation:**

A 9-year-old boy presented with a headache, and ultrasonography, computed tomography, and magnetic resonance imaging revealed a heterogeneous enhancement of soft tissue originating from the upper pole of the left kidney, measuring approximately 11.0 × 10.0 × 8.0 cm. A left radical nephrectomy was performed using an intraperitoneal approach through an anterior subcostal incision. Pathological examination suggested clear cell sarcoma of the kidney or synovial sarcoma of the kidney based on morphological and immunohistological features. Using genetic analysis, a final diagnosis of spindle cell pattern clear cell sarcoma of the kidney was made based on the absence of the *SYT-SSX* fusion gene. After adjuvant chemo-radiotherapy was administered, no recurrence or metastasis has been identified as of 60 months postoperatively.

**Conclusion:**

In this case, it was difficult to discriminate clear cell sarcoma of the kidney from synovial sarcoma of the kidney based on histopathological examination alone, and genetic analysis was required. Accurate pathological diagnosis of pediatric renal tumor is important for determining optimal treatment and preventing recurrence and metastasis.

## Background

Clear cell sarcoma of the kidney (CCSK) is a rare malignant renal tumor that primarily occurs in children and was first described by Kidd et al. in 1970 [1]. CCSK has recently been regarded as a malignancy distinct from Wilms tumor, and it has been classified as a tumor with unfavorable histology, along with synovial sarcoma of the kidney (SSK) [2]. SSK is also a rare malignant renal tumor, and was first described by Argani et al. in 2000 [2]. CCSK and SSK are difficult to differentiate pathologically from Wilms tumors, sarcomatoid renal cell carcinoma, and undifferentiated carcinoma. However, a precise diagnosis is important, as these unique tumors require different treatment regimens.

Herein we describe our experience with a 9-year-old boy diagnosed with CCSK that was difficult to discriminate from SSK.

## Case presentation

**Figure 1:**
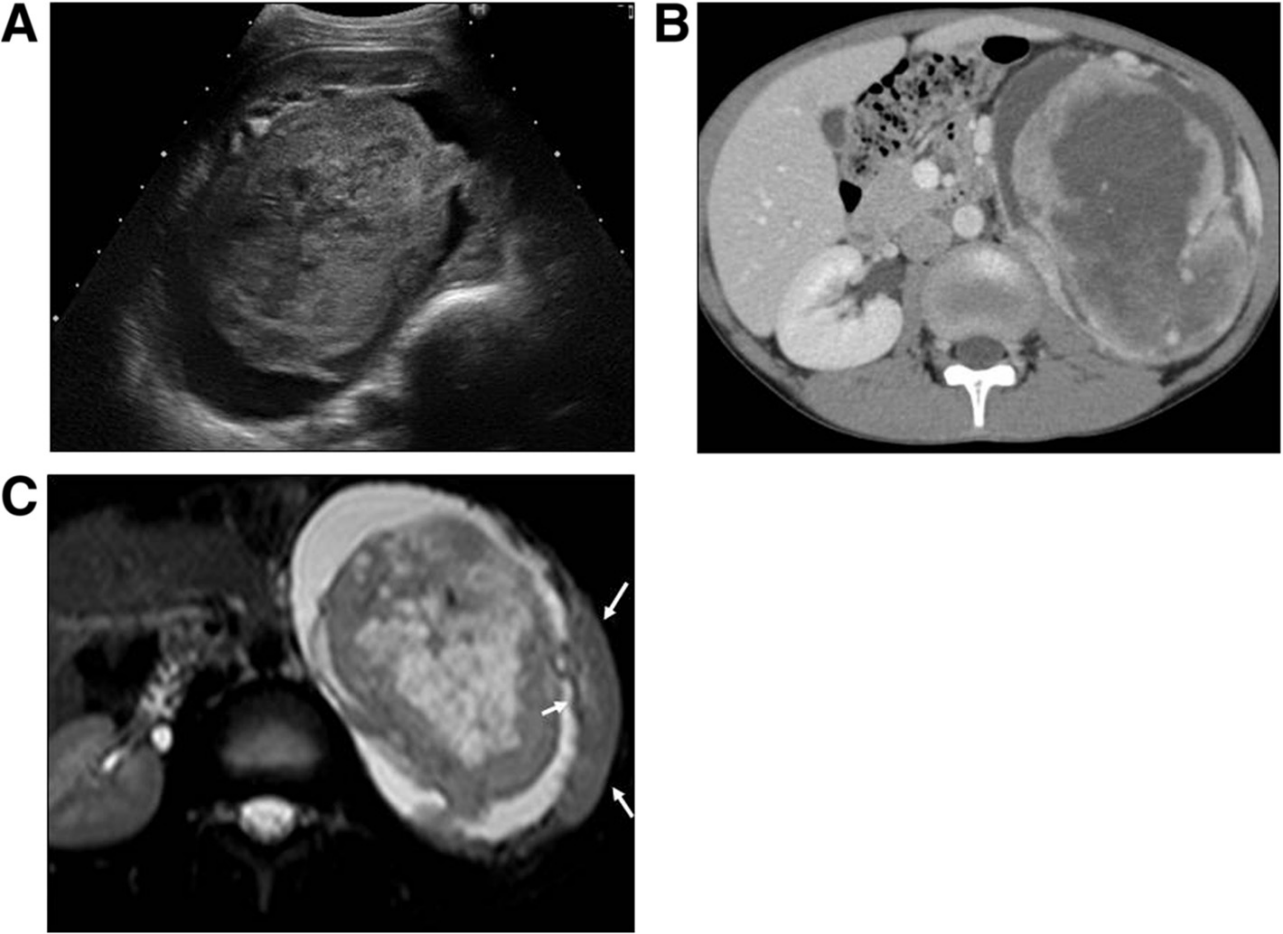
**Images of the left kidney tumor. (A)** Ultrasonography showing a solid tumor in the left kidney. The tumor borders were clear, and the tumor measured 11 × 10 × 8 cm. We detected mixed echogenicity inside the tumor. **(B)** Contrast-enhanced computed tomography showing a solid tumor with clear borders and contrasted disproportionate, with no lymph node swelling or tumor embolism. **(C)** Magnetic resonance imaging (T2-weighted) showing necrosis and hemorrhage inside the tumor. The tumor was suspected to have originated from left kidney tissue (arrow; normal kidney).

**Figure 2:**
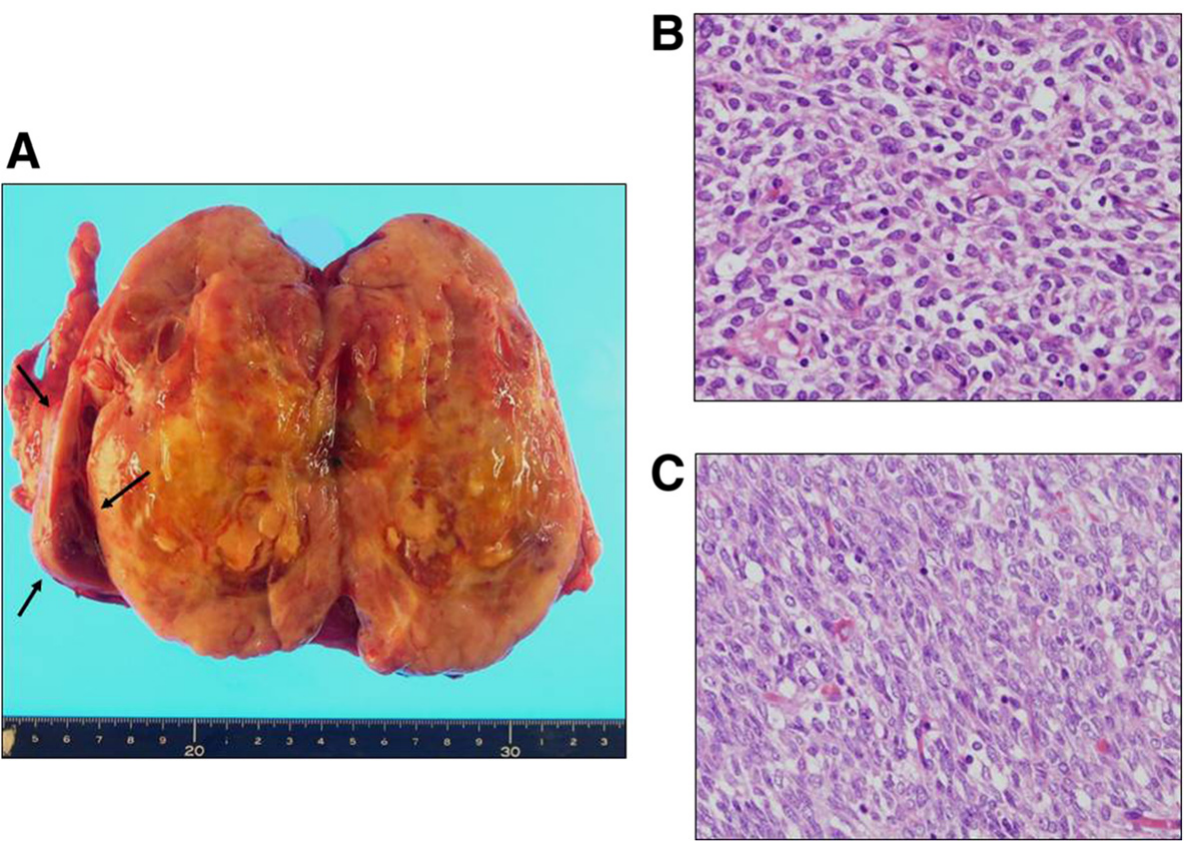
**Removed kidney and pathology of the tumor. (A)** The tumor was solid, elastic soft, and light yellow in coloration, with degenerative necrosis, hemorrhage and cystic lesion apparent inside the tumor. The resected kidney weighed 630 g. The borders of the normal kidney were clear (arrow: normal kidney). **(B)** This part of the tumor exhibited clear cells with scant cytoplasm and circular. Nuclei, suggesting clear cell sarcoma of the kidney (CCSK). Hematoxylin-eosin staining (reduced from × 400). **(C)** This part of the tumor showed spindle-shaped cells with a comparatively high nucleus to cytoplasm ratio. These findings suggested SSK or spindle-pattern CCSK. Hematoxylin-eosin staining (reduced from × 400).

A 9-year-old boy presented with a headache; he was treated for a common cold, but his symptoms did not improve. Further examinations identified that he had high blood pressure. Ultrasonography (Figure 1A), computed tomography (Figure 1B), and magnetic resonance imaging (Figure 1C) revealed heterogeneous enhancement of soft tissue originating from the upper pole of the left kidney, measuring approximately 11.0 × 10.0 × 8.0 cm. Further evaluation including bone scan did not demonstrate any evidence of metastasis. A left radical nephrectomy was performed using an intraperitoneal approach through an anterior subcostal incision. The tumor was solid, although degenerative necrosis and hemorrhage were observed inside the tumor (Figure 2A).

**Figure 3:**
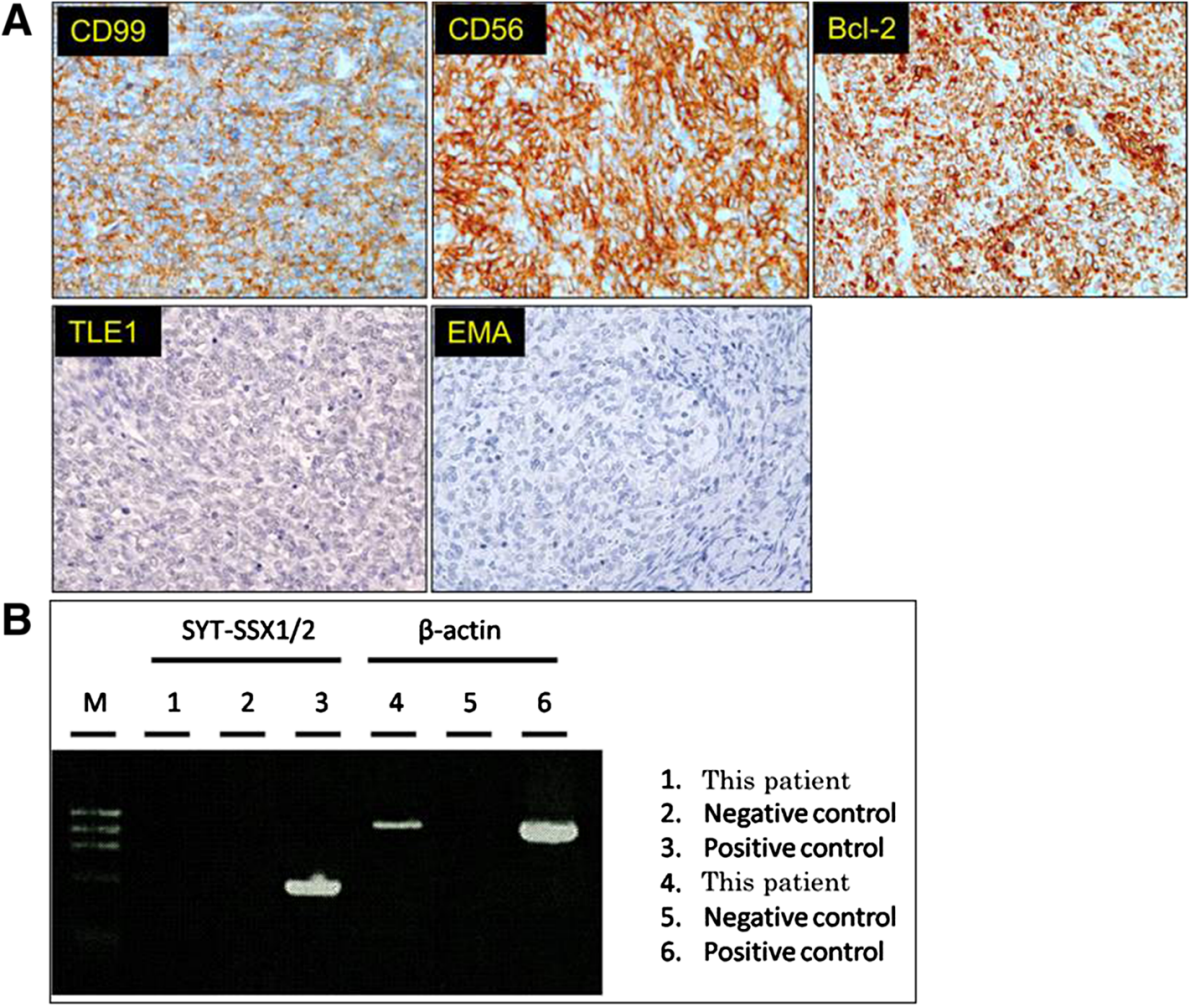
**Immunohistological staining for Bcl-2, CD-56, CD-99, TLE1, and EMA and polymerase chain reaction of** ***SYT-SSX*** **. (A)** Positive immunohistological staining for Bcl-2, CD-56, and CD-99 (reduced from × 400). Negative immunohistological staining for TLE1, and EMA (reduced from × 400). **(B)** No *SYT-SSX* fusion gene was detected in tumor tissue by polymerase chain reaction.

Pathological examination revealed a high nucleus to cytoplasm ratio, proliferation of short, spindle-shaped tumor cells (Figure 2B), and the presence of clear cells with scant cytoplasm (Figure 2C). Staining for cytokeratin yielded negative results, while tumor cells were positive for vimentin, Bcl-2, CD-56, and CD-99 (Figure 3A). Differential diagnosis suggested CCSK or SSK; however, a final diagnosis of spindle cell pattern CCSK was made based on the absence of the *SYT-SSX* fusion gene by polymerase chain reaction (Figure 3B) and the negative results for transducin-like enhancer of split 1 (TLE1) and epithelial membrane antigen (EMA) staining (Figure 3A). The final pathological stage was T3aN0M0 stage 1, according to the updated National Wilms Tumor Study-5 definition [3].

Concomitant chemo-radiotherapy was administered. The patient underwent adjuvant radiotherapy to the left nephrectomy bed (1080 cGy). As adjuvant chemotherapy, vincristine, adriamycin, etoposide, and cyclophosphamide were administered 1 day every 4 weeks over a 6-month period, in accordance with the Japan Wilms Tumor Study protocol [4].

No serious therapy-related side effects were observed. No evidence of local recurrence or metastasis has been identified as of 60 months following the operation.

### Discussion

In this case, CCSK was difficult to discriminate from SSK. Differentiating CCSK from SSK, primitive neuroendocrine tumor of the kidney, and sarcomatoid renal cell carcinoma remains difficult [5-8]. No clinical or imaging characteristics can aid a definitive preoperative diagnosis; thus, a diagnosis of CCSK always requires pathological confirmation.

Pathologically, CCSK is characterized by tumor cells with fine nuclear chromatin, pale cytoplasm, and indistinct cell borders, these cells form nests separated by fibrovascular stroma [5]. However, CCSK has multiple variant patterns, including myxoid, sclerosing, cellular, epithelioid, palisading, spindle-cell, storiform and anaplastic patterns [5]. SSK consists of plump spindle cells in a mono- or biphasic pattern with minimal cytoplasm and active mitotic figures [8]. In addition, cystic lesions are commonly present and are lined with epithelial cells that possess eosinophilic cytoplasm with apical nuclei, resulting in a hobnail appearance [8].

Immunohistological staining can help to differentiate CCSK from SSK, as CCSK typically stains positive for vimentin and negative for cytokeratin, Bcl-2, CD-56, and CD-99 [5,6]. However, Bcl-2, CD-56, and CD-99 may be expressed in some cases. In the case of SSK, specimens are typically positive for cytokeratin, vimentin, Bcl-2, CD-56, CD-99, TLE1, and EMA, and are negative for actin, desmin, S-100, and CD-34 [8-10].

Discriminating between SSK and CCSK based on morphological findings is often easy, though it can be difficult when spindle-form cells are present. In addition, immunohistochemical staining sometimes shows atypical results. However, in genetic examinations, polymerase chain reaction testing has greatly aided the diagnosis of SSK by allowing detection of the *SYT-SSX* fusion gene that results from translocation of the *SYT* gene on chromosome 18 with the *SSX* gene on the X chromosome [11,12]. No *SYT-SSX* fusion gene is present in CCSK, though the chromosomal translocation t(10;17)(q22;p13) is recognized in 12% of CCSK cases [13]. But, translocation t(10;17)(q22;p13) is not recognized in this case.

In the present case, we observed a high nucleus to cytoplasm ratio and an increased presence of spindle-shaped tumor cells and clear cells with scant cytoplasm. On immunohistochemical staining, tissues stained positive for vimentin, Bcl-2, CD-56, and CD-99, but were negative for cytokeratin, TLE1, and EMA. CCSK and SSK were potential diagnoses based on the pathological examination, but a final diagnosis of spindle -cell CCSK was made based on the absence of the *SYT-SSX* fusion gene.

For CCSK treatment, surgery, radiotherapy, and chemotherapy are treatment options that may be administered separately or in combination. Benchekroun et al. reported the case of a patient who underwent surgery followed by combination chemotherapy with cisplatin and doxorubicin; no metastases developed over the course of 4 years, whereas metastases occurred within months in two patients who did not receive postoperative chemotherapy or radiotherapy [14]. In addition, Bhayani et al. noted that a patient treated with surgery and combination chemotherapy (actinomycin, vincristine and doxorubicin) remained disease-free at 24 months postoperatively [15]. The National Wilms’ Tumor Study-5 trial showed a 4-year overall survival rate of 75% [16]. While surgical resection and ifosfamide-based chemotherapy represent the mainstays of SSK management, optimal treatment remains unclear [8,17]. SSK exhibits an aggressive clinical course with poor outcomes. Of the first 20 cases reported, 7 patients showed local or metastatic recurrence of the disease and two patients have died [18].

In the future, pathological and genetic information is likely to influence guidelines regarding treatment and follow-up in patients with CCSK and SSK. Further studies with a larger number of CCSK and SSK cases are needed to provide more knowledge regarding the diagnosis of pathological types and appropriate treatments.

## Conclusion

We report herein the case of a 9-year-old boy who underwent radical nephrectomy for a tumor in the left kidney. A final diagnosis of CCSK was made based on genetic analysis, and adjuvant chemo-radiotherapy was administered. Accurate pathological diagnosis of pediatric renal tumor is important for determining optimal treatment and preventing recurrence and metastasis.

## Consent

Written informed consent was obtained from the patient’s parents for publication of this Case Report and any accompanying images. A copy of the written consent is available for review by the Editor-in-Chief of this journal.

## Abbreviations

CCSK: Clear cell sarcoma of the kidney
SSK: Synovial sarcoma of the kidney
TLE1: Transducin-like enhancer of split 1
EMA: Epithelial membrane antigen
